# Rail Road Killed and Wounded

**Published:** 1865-02

**Authors:** 


RAIL ROAD KILLED AND WOUNDED.—There were more people killed and wounded by railroad accidents last year than any year since 1854. One hundred and forty accidents occurred; four hundred and four lives were lost, and one thousand eight hundred and forty-six persons were wounded.

